# Progress in the clinical use of bone marrow aspirate concentrate for knee osteoarthritis: an expert opinion

**DOI:** 10.1186/s13018-025-06509-1

**Published:** 2025-12-10

**Authors:** Filippo Migliorini, Marco Pilone, Francesco Simeone, Madhan Jeyaraman, Andreas Bell, Nicola Maffulli

**Affiliations:** 1 Department of Trauma and Reconstructive Surgery, University Hospital of Halle, Martin-Luther University Halle-Wittenberg, Ernst-Grube-Street 40, 06097 Halle (Saale), Germany; 2 Department of Orthopaedics and Trauma Surgery, Academic Hospital of Bolzano (SABES-ASDAA), 39100 Bolzano, Italy; 3 Residency Program in Orthopaedics and Traumatology, University of Milan, Milan, Italy; 4 Department of Orthopaedics, ACS Medical College and Hospital, Dr MGR Educational and Research Institute, Chennai, Tamil Nadu 600077 India; 5 Department of Orthopaedic and Trauma Surgery, Eifelklinik St. Brigida, 52152 Simmerath, Germany; 6 Department of Trauma and Orthopaedic Surgery, Faculty of Medicine and Psychology, University La Sapienza, 00185 Roma, Italy; 7 School of Pharmacy and Bioengineering, Keele University Faculty of Medicine, Stoke on Trent, ST4 7QB UK; 8 Centre for Sports and Exercise Medicine, School of Medicine and Dentistry, Barts and the London, Mile End Hospital, Queen Mary University of London, London, E1 4DG UK

**Keywords:** Knee osteoarthritis, Biological derivatives, Bone marrow aspirate concentrate, BMAC

## Abstract

Knee osteoarthritis (OA) is a common degenerative joint condition and a major cause of disability. Orthobiological therapies aim to regenerate articular cartilage and delay or stop the progression of the degenerative lesion. Intra-articular injections of biological derivatives have been increasingly used in the last decade, although the indications for using bone marrow aspirate concentrate (BMAC) are still unclear. The present expert opinion reviewed the current literature on BMAC in the management of knee OA, providing an update on the current indications for the selection of the ideal patient, as well as the preparations and efficacy of BMAC compared to other biological alternatives. Clinical studies that investigated BMAC in the management of knee OA were identified and discussed. BMAC is a valuable source of mesenchymal stem cells, offering potential benefits in attenuating the inflammatory pathway associated with knee OA. Intra-articular administration of BMAC has shown effectiveness in clinical trials, improving the functional outcomes of patients. However, the superiority of BMAC over other orthobiologic treatments cannot be assessed, given the conflicting results presently available.

## Introduction

Knee osteoarthritis (OA) is common [1–3]. Approximately one-third of the adult population reports signs of joint degeneration, and 8.9% symptomatic OA [4–6]. Female sex, advanced age, occupation, higher body mass index (BMI), traumas, varus/valgus deformity and participation in high-impact sports are risk factors for knee OA [7–9]. In OA, mechanosensitive and biochemical pathways are activated, resulting in deformation of the joint [8, 10–13]. Articular cartilage loss, osteophytes, synovial inflammation, endochondral ossification with vascular penetration, and subchondral bone cysts are common in knee OA [2, 14–18]. Clinically, impaired knee function, stiffness, reduced range of motion, muscle weakness, and persistent pain are common, leading to long-term consequences such as reduced physical activity, deconditioning, impaired sleep, depression, and disability [19–21]. Conservative management, such as non-steroidal anti-inflammatory drugs, glucosamine, chondroitin sulphate, omega 3 fatty acids, hyaluronic acid and corticosteroids intra-articular injections, is only palliative and does not prevent the progression of OA to surgical management [22–25]. Orthobiological therapy aims to regenerate the articular cartilage, slowing or stopping degeneration progression [26–30]. Intra-articular injections of biological derivatives have become common in the last decade [31–33]. Platelet-rich plasma (PRP) is obtained following centrifugation of autologous blood, with subsequent release of cytokines and growth factors concentrated in platelet granules [34, 35]. Mesenchymal stem cell sources include adipose tissue (ADSC), human umbilical cord blood mesenchymal stem cells (MSCs), and bone marrow aspirate concentrate (BMAC) [36–38]. BMAC contains a heterogeneous mixture of cells with different functions: regenerative repair, immunomodulation and trophic factor release [39, 40]. Only 0.001–0.01% of BMAC content are mesenchymal stem cells, predominantly hematopoietic, with platelets, various hematopoietic cells at various stages of differentiation and cytokines [41, 42]. Hematopoietic progenitors can morph into mesenchymal stem cells and differentiate into chondrocytes, allowing adequate cartilage repair [43, 44]. The current indications for the effective application of BMAC are still unclear. The present expert opinion reviewed the current evidence on BMAC in the management of knee OA, giving an update on the current indications to select appropriate patients and the preparations and efficacy of the treatment compared to other biological alternatives.

## Methods

### Search strategy

A comprehensive literature search was conducted to identify all clinical investigations evaluating BMAC for the management of knee OA. Only articles published in peer-reviewed journals were considered. Studies published in English, German, Italian, French, and Spanish were eligible, reflecting the authors’ language proficiency. Studies with levels of evidence I to III, according to the Oxford Centre for Evidence-Based Medicine [45], were included. In October 2025, PubMed, Web of Science, and Scopus were systematically searched. The medical subject headings (MeSH) used in each database are reported in Table 1.

**Table 1:** Strings used for the search in each database (WoS: web of Science)

PubMed	(“Bone Marrow Aspirate Concentrate“[Title/Abstract] OR BMAC[Title/Abstract] OR “bone marrow concentrate“[Title/Abstract] OR “bone marrow-derived“[Title/Abstract] OR “bone marrow cells“[Title/Abstract] OR “mesenchymal stem cells“[Title/Abstract] OR MSC[Title/Abstract]) AND (“knee osteoarthritis“[MeSH Terms] OR “knee osteoarthritis“[Title/Abstract] OR “knee OA“[Title/Abstract] OR “knee joint“[Title/Abstract] OR “cartilage repair“[Title/Abstract] OR “orthobiologic“[Title/Abstract])
Scopus	TITLE-ABS-KEY(“bone marrow aspirate concentrate” OR BMAC OR “bone marrow concentrate” OR “bone marrow-derived" OR “bone marrow cells” OR “mesenchymal stem cells” OR MSC) AND TITLE-ABS-KEY(“knee osteoarthritis” OR “knee OA” OR “knee joint" OR “cartilage repair” OR “orthobiologic”)
WoS	TS=(“bone marrow aspirate concentrate” OR BMAC OR “bone marrow concentrate” OR “bone marrow-derived" OR “bone marrow cells” OR “mesenchymal stem cells” OR MSC) AND TS=(“knee osteoarthritis” OR “knee OA” OR “knee joint" OR “cartilage repair” OR “orthobiologic”)

## Results

### Study selection

**Fig. 1:**
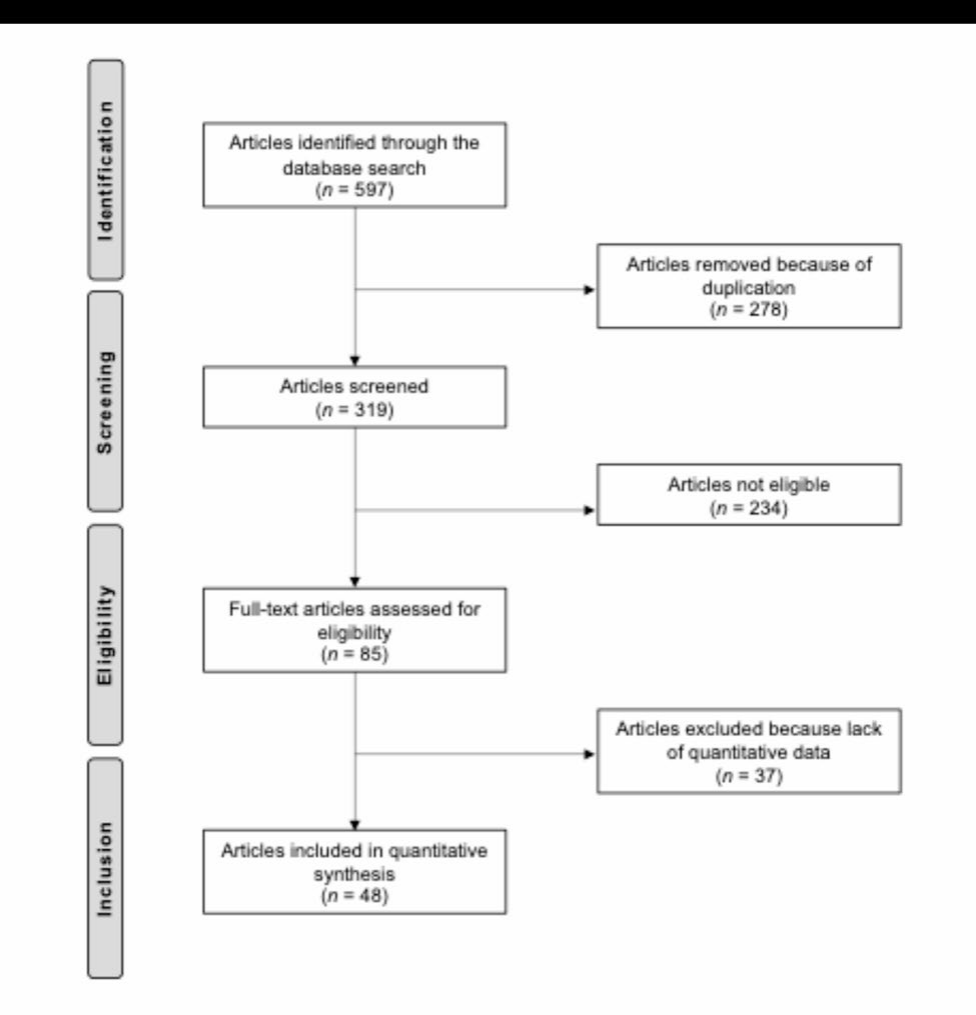
PRISMA flow chart of the literature search

The initial search across the selected databases yielded 597 records. After removing 278 duplicates, 319 unique articles remained for title and abstract screening. Of these, 234 papers were excluded as they did not meet the inclusion criteria. The most common reasons for exclusion were unsuitable study design (*n* = 142), low level of evidence (*n* = 46), interventions not related to bone marrow aspirate concentrate or knee osteoarthritis (*n* = 39), and language limitations (*n* = 7). A full-text assessment was then performed for the remaining 85 articles. After detailed evaluation, 37 studies were excluded because of incomplete, non-quantitative, or irrelevant outcome data. In the end, 48 clinical studies were included in the qualitative synthesis and discussed in this review. The flow of study selection is reported in Fig. 1.

## Expert opinion

The use of stem cells to enhance tissue healing has recently gained considerable attention, given their potential to heal and regenerate tissues [46]. Stem cells, characterised by their self-renewal ability and inherent plasticity, can differentiate into various cell types depending on their biological environment [47]. Unlike embryonic stem cells, which can differentiate into all three primary germ layers, multipotent adult stem cells can differentiate into a specific germ layer [48]. For example, MSCs can differentiate into bone, tendon, cartilage, or muscle [49]. BMAC is a known reservoir of MSCs and growth factors [31, 39]. The predominant mechanism responsible for the beneficial effects of stem cells involves paracrine signalling, in which the cells modulate the local environment to enhance healing and repair, rather than directly engaging in tissue regeneration [50]. MSCs and growth factors modulate the inflammatory pathway associated with OA. The differentiation of MSCs into chondrocytes is influenced by various cofactors, including insulin, selenium, and transferrin, with TGF-β being the most crucial [51]. TGF-β plays a pivotal role in type II collagen production, T-lymphocyte differentiation, and the regulation of metalloprotease activity levels [52, 53]. In addition, MSCs exert immunomodulatory effects by downregulating pro-inflammatory cytokines such as IL-1β and TNF-α, while upregulating anti-inflammatory mediators including IL-10 and TGF-β [54, 55]. These paracrine interactions influence macrophage polarisation and reduce synovial inflammation, which is thought to contribute to pain relief and improved joint homeostasis in osteoarthritis [56].

### Harvesting

Various harvesting sites are accessible, including the anterior and posterior iliac crests, the ilium, the proximal humerus, the proximal tibia, the distal femur, the distal tibia, the sternum, the mandible, and the calcaneum [46, 57, 58]. The recommended harvesting site is the anterior iliac crest. However, the posterior iliac crest is the safest site for harvesting because of its thickness [59], with no difference in MSCs concentration between the anterior and posterior iliac crest [60]. The number of MSCs obtained from the tibial plateau was approximately half of those gathered from the iliac crest [60]. No consensus exists on the best BMAC harvesting techniques, and various devices and protocols have been used across studies. Peters et al. [61] favour multiple insertions (up to four) because of the most appropriate BMAC volume and concentration, while Oliver et al. [62] argue that no difference in BMAC volume and concentration exists between single and multiple insertions, and the single insertion is less painful [61]. Schäfer et al. [63] and Dragoo et al. [64] demonstrated a statistically significant difference in BMAC composition using different extraction tools. When using a single processing system, the amount of MSCs harvested does not depend on the patient’s sex, age and BMI [65]. Differences in BMAC preparation protocols, including centrifugation speed, duration, and volume ratios, can significantly influence the final cell composition and cytokine concentration, potentially affecting clinical outcomes [66]. Standardisation of these parameters remains lacking, which may contribute to the variability observed across published studies.

### Site of injection

Intra-articular injection of BMAC could reduce the inflammation in synovium and articular cartilage by suppressing the NFkB pathway [67, 68]. The rapid pain relief after the injection is attributed to the interaction with the cannabinoid receptors on the synovial cells [69]. Shoukrie et al. [70] conducted a systematic review that included 10 studies, including 6 RCTs, on intra-articular injection of BMAC. A statistically significant increment in function was found at 6, 12 and 24 months compared to the baseline [70]. The MRI evaluation did not show any progression of the cartilage defects after 6 months of follow-up [70]. The longest follow-up study [71] analysed the functional outcomes in 55 patients for 5 years. VAS score, Tegner scale and WOMAC scale showed a statistically significant improvement after intra-articular injection of BMAC [71]. The authors emphasised the potential positive impacts on younger patients, given the mean age of 45.3 ± 9.6 years [71]. Shapiro et al. [72] compared the outcomes in patients with bilateral knee OA who randomly received BMAC in one knee and a placebo in the other. No statistically significant difference was observed in pain and quality of life scores after 12 months of follow-up [72]. Both showed a decrease in pain and an increase in quality of life scores compared to baseline [72]. MRI showed no significant change in the cartilage defects after six months [72]. The “homing effects” of MSCs could affect these results [73]. This mechanism was observed in models with intravascular injections and cannot be broadly extrapolated [74]. Nonetheless, increasing evidence suggests MSCs and growth factors may migrate from the injection site into the general circulation [75]. According to recent reviews, the multistep homing cascade of MSCs involves chemokine-receptor signalling (e.g., SDF-1/CXCR4), selectin- and integrin-mediated tethering, trans-endothelial migration, and may include transplantation into injured tissue [76, 77]. However, the actual engraftment rate after local or systemic delivery remains low. A landmark intra-articular MSC injection study found that labelled MSCs were present at the defect site at one day and one week, but none were detected beyond one month [78]. Similarly, Huang et al. [54] emphasised that while homing is theoretically plausible, most therapeutic effects are likely mediated by transient paracrine signalling rather than permanent cell engraftment. Clinically, this implies that, despite potential MSC migration, durable localisation and differentiation of MSCs at the graft or tunnel site remain unproven, underscoring that the beneficial effects of MSC/BMAC therapies are likely driven by secreted factors rather than by proper tissue integration [79, 80].

In patients with knee OA, the number of MSCs in synovial fluid is increased [81], whereas the number in subchondral bone marrow is decreased [82]. This has led to differing opinions on whether to opt for intra-articular or subchondral injection of BMAC [83]. Hernigou et al. [84] conducted an RCT comparing intra-articular and subchondral BMAC injections in 60 patients with bilateral knee OA. After 2 years of follow-up, MRI showed a slower progression of OA in the subchondral injection group than in the intra-articular injection group [84]. After 15 years of follow-up, 20% of the knees in the subchondral group and 42% of the knees in the intra-articular group had undergone TKA [84]. Subchondral BMAC injection showed better results in young patients with severe OA secondary to corticosteroid-induced osteonecrosis [81]. 60 knees of 30 patients with bilateral secondary OA were randomly treated with TKA and subchondral BMAC. After 12 years of follow-up, the Knee Score showed no statistically significant difference between the two groups [81]. Of the 30 knees treated with BMAC, only 3 needed a TKA [81]. Kon et al. [85] analysed the combined intra-articular and subchondral injection of BMAC. After 2 years of follow-up, plain radiographs did not show any signs of OA progression, and MRI showed a significant reduction of bone marrow oedema [85].

Gobbi et al. [86] investigated long-term clinical outcomes of cartilage repair using a hyaluronic acid-based scaffold embedded with BMAC. The mean cartilage defect was 6.5 cm^2^. Functional scores showed statistically significant improvements after six years of follow-up [86]. Patients below 45 demonstrated superior outcomes, indicating that the ideal candidates for this treatment are younger patients with medium-sized chondral defects [86]. Despite these promising results, a small sample size limited the study, and no MRI follow-up was conducted.

### Orhobiologics

Another source of MSCs is adipose tissue (MFAT) [87]. One MSC can be derived from every 100 adipose cells, whereas the ratio is 1 MSC for every 100,000 bone marrow cells [14]. The cells are not negatively affected by ageing, which is particularly advantageous for the elderly [88]. Employing adipose-derived MSCs is linked with minimal side effects, and prior studies have shown no complications related to malignancy or cancer [89]. Pintore et al. [31] analysed 51 patients who received BMAC and 51 patients who received MFAT. A statistically significant improvement in KOOS, OKS and VAS was observed in both groups compared to baseline [31]. No difference was seen between the two groups [31]. Patients with mild OA (Kellgren and Lawrence II) showed better functional and clinical outcomes than patients with severe OA [31]. Similar results were obtained in.

41 patients treated with BMAC and 35 patients treated with MFAT showed improvement in clinical and functional scores compared with baseline, with no difference between the two groups [90]. The existing data do not permit assessing the superiority of BMAC over MFAT.

Several studies compared BMAC with hyaluronic acid (HA) and platelet-rich plasma (PRP) [90–93]. In an RCT on 175 patients [94], 111 were treated with BMAC, 30 with HA and 34 with PRP. The BMAC group exhibited better clinical and functional outcomes than the HA and PRP groups after 12 months of follow-up [94]. No difference was found between HA and PRP [94]. Another RCT [95] did not confirm the superiority of BMAC over PRP. No difference in functional parameters was found between the BMAC and PRP groups after 2 years of follow-up [95]. The long-term efficacy of BMAC over HA is questioned [96]. A statistically significant difference in KOOS score was not observed after 12 months of follow-up between the two groups [96]. VAS was lower in the BMAC group than in the HA group [96]. Boffa et al. [97] confirmed no difference in functional scores between BMAC and HA. In individuals with mild OA, the VAS score showed a statistically significant improvement in the BMAC group compared to the HA group. A recent meta-analysis [98], including only level I studies, compared BMAC, HA, and PRP. Patients who received BMAC showed better WOMAC, IKCD, and VAS scores than those who received HA [98]. No statistically significant difference was found between the PRP and BMAC groups [98]. Another meta-analysis confirmed these results on a larger number of studies (level I and II) [99]. BMAC injections produced the best pain relief after 12 months of follow-up [99]. BMAC has a significantly higher level of IL-1Ra than LR-PRP and LP-PRP. The anti-inflammatory biological effect of IL-1Ra can explain the long-term pain relief after BMAC injection [100].

### Future prospective

The use of BMAC garnered significant attention for enhancing patients’ quality of life by relieving pain and improving knee function, potentially delaying the need for invasive surgical options and offering a valuable alternative for managing knee conditions and improving patient well-being. The existing published studies employ varied methodologies to assess the efficacy of BMAC treatment. Several BMAC formulations, patient profiles, and OA grades were included, but there was insufficient clarity regarding OA severity. Short follow-up periods and limited comparisons with other orthobiologic treatments hinder the determination of clinical superiority. Larger-scale, longer-term studies are imperative to establish proper indications for BMAC, particularly regarding its use in early-stage OA for young versus elderly patients. The regenerative potential of BMAC remains inadequately demonstrated, necessitating additional preclinical and MRI studies to evaluate its regenerative properties comprehensively. Clarifying these aspects is pivotal to advancing the clinical application of BMAC and optimising its effectiveness in managing OA across diverse patient populations and disease stages. Further research is essential to refine treatment protocols, enhance understanding of the regenerative capabilities of BMAC, and ultimately establish evidence-based guidelines for its clinical use.

## Conclusion

Intra-articular injections of BMAC may offer benefits for modulating knee inflammation in OA. Whether BMAC performs better than other orthobiologics is still unclear, and additional high-quality investigations are strongly required.

## Data Availability

Data is provided within the manuscript.
